# Galectin-3 as a Marker for Increased Thrombogenicity in COVID-19

**DOI:** 10.3390/ijms24097683

**Published:** 2023-04-22

**Authors:** Marianna Puccini, Kai Jakobs, Leander Reinshagen, Julian Friebel, Philipp-Alexander Schencke, Emily Ghanbari, Ulf Landmesser, Arash Haghikia, Nicolle Kränkel, Ursula Rauch

**Affiliations:** 1 Deutsches Herzzentrum der Charité, Department of Cardiology, Angiology and Intensive Care Medicine, 12203 Berlin, Germany; 2 DZHK (German Center for Cardiovascular Research), Partner Site Berlin, 10178 Berlin, Germany; 3 Berlin Institute of Health at Charité—Universitätsmedizin Berlin, 10178 Berlin, Germany

**Keywords:** galectin-3, increased thrombogenicity, platelet activation, COVID-19

## Abstract

Galectin-3 is a beta-galactoside-binding lectin involved in inflammation and lung fibrosis and postulated to enhance thrombosis. In COVID-19, it is considered to be a prognostic marker of severity. The aim of this study was to evaluate whether galectin-3 is associated with thrombogenicity in COVID-19. Patients with moderate-to-severe COVID-19 (COVpos; n = 55) and patients with acute respiratory diseases, but without COVID-19 (COVneg; n = 35), were included in the study. We measured the amount of galectin-3, as well as other platelet and coagulation markers, and correlated galectin-3 levels with these markers of thrombogenicity and with the SOFA Score values. We found that galectin-3 levels, as well as von Willebrand Factor (vWF), antithrombin and tissue plasminogen activator levels, were higher in the COVpos than they were in the COVneg cohort. Galectin-3 correlated positively with vWF, antithrombin and D-dimer in the COVpos cohort, but not in the COVneg cohort. Moreover, galactin-3 correlated also with clinical disease severity, as measured by the SOFA Score. In patients with acute respiratory diseases, galectin-3 can be considered as a marker not only for disease severity, but also for increased hypercoagulability. Whether galectin-3 might be a useful therapeutic target in COVID-19 needs to be assessed in future studies.

## 1. Introduction

The coronavirus disease 2019 (COVID-19) caused by severe acute respiratory syndrome coronavirus type 2 (SARS-CoV-2), which was declared as a pandemic in March 2020, has affected over 650 million people worldwide, and approximately 6.8 million deaths have been attributed to the disease by the WHO [1].

In addition to a state of hyperinflammation [2,3], from the beginning of the pandemic, it was observed that COVID-19 patients displayed a higher thrombotic and thromboembolic risk [4,5]. The different pathophysiological mechanisms involved in COVID-19-related coagulopathy include endothelial dysfunction, platelet hyperreactivity, neutrophil extracellular traps and complement system activation, among others [5,6,7]. There are several thrombogenicity markers (including markers for platelet activation, coagulation and fibrinolysis) that have been observed to play a role in the pathophysiology of COVID-19. First, an increase in von Willebrand Factor levels is seen, and this has been associated with the development of immunothrombosis in COVID-19 [8]. Other markers of platelet hyperreactivity, such as MPV or P-selectin, have also been associated with COVID-19 severity [7,9,10].

Antithrombin is a protein that regulates the activity of several coagulation factors [11], acting as an anticoagulant [12], and therefore, playing an important role in clot degradation. D-dimer is a fibrin degradation product that is used as a biomarker of coagulation, and also, of fibrinolysis [5]. It is associated with the severity of a hypercoagulable state [13]. Since the beginning of the COVID-19 pandemic, elevated D-dimer levels were discovered in approximately 10% of COVID-19 patients and associated with a worse prognosis [10,14].

Tissue plasminogen activator (tPA) is one of the molecules that is involved in the regulation of fibrinolysis by activating this system [15]. It has been observed that the amount of tPA is higher in COVID-19 patients in comparison to that in healthy controls and is also associated with a higher mortality rate [16].

Galectin-3 is a ß-galactoside-binding lectin with a number of regulatory effects on cellular growth, cellular differentiation, adhesion and tissue repair [17]. It has also been described to be involved in the pathogenesis of different conditions, such as chronic inflammation, atherosclerosis as well as cardiovascular disease [18,19], and has been recommended as a biomarker for heart failure [20,21].

Galectin-3 is a prognostic marker for COVID-19 severity [22,23,24]. Moreover, it contributes to the development of lung fibrosis in COVID-19, as well as in other pulmonary diseases [25,26,27].

Recently, Chen et al. described the crucial role of galectin-3 in platelet activation and thrombus formation in patients with coronary artery disease [28,29]. However, the exact involvement of galectin-3 in the pathophysiology of thrombotic disease in COVID-19 is yet unknown.

Furthermore, it has been described by Shahneh et al. that regulatory T cells (Treg) can accumulate in thrombi and play an important role in blood clot resolution [30]. One of the suppressive immunoregulatory cytokines that is secreted by Treg is interleukin-10 (IL-10) [31]. Although IL-10 is known for its immunomodulatory effects, it has also been seen to be elevated in COVID-19 patients and associated with COVID-19 severity [32,33]. This has been interpreted as negative feedback to counter-regulate hyperinflammation [32,34]. Another typical characteristic of Tregs is the expression of the IL-2 receptor (CD25) [31,35,36]. IL-2, although it is not secreted by Tregs, plays an important role in the differentiation of these cells [31,36]. In COVID-19, IL-2 is one of the signature interleukins of the exaggerated inflammatory response that has been observed in patients with a more severe disease [37,38].

On one hand, we therefore aimed to explore a possible association of galectin-3 with other markers of an increased thrombotic risk in patients with COVID-19. We further aimed to evaluate if there is a correlation between galectin-3 and markers associated with Treg in COVID-19.

## 2. Results

### 2.1. Baseline Characteristics

The study included a total of 90 hospitalized patients, of which 55 were SARS-CoV-2-positive ones (COVpos) and 35 were patients had an acute respiratory infection other than COVID-19 (COVneg). The baseline characteristics of the population are shown in Table 1. The median age was 70 years, and 64.4% of the total population were male. More patients in the COVpos cohort died during hospitalization in comparison to the number of COVneg patients who died. More patients in the control group had heart failure, coronary artery disease, peripheral artery disease and chronic obstructive pulmonary disease (COPD). The use of medication was comparable between the groups, except for a higher use of betablockers in the COVneg group and of glucocorticoids in the COVpos group (Table 1). The laboratory parameters were also not statistically different between both study populations.

### 2.2. Galectin-3 Levels Are Higher and Correlate with Markers of Increased Thrombogenicity in Patients with COVID-19

**Figure 1:**
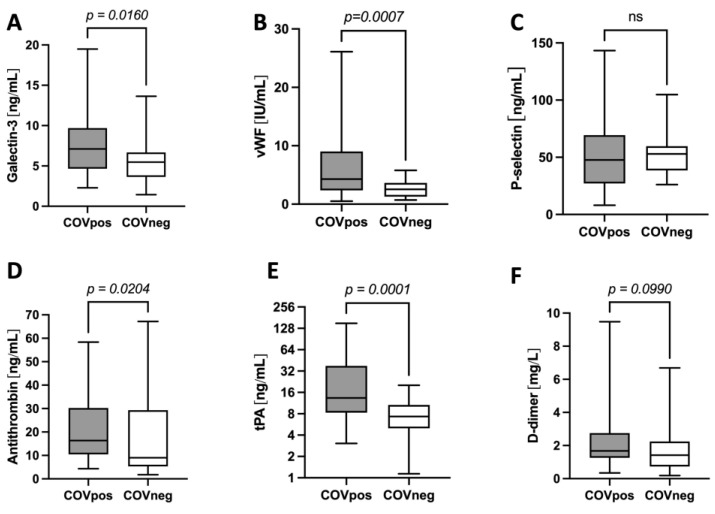
Levels of galectin-3 (**A**) and markers of increased thrombogenicity (**B**–**F**) in COVpos (n = 55) and COVneg (n = 35) patients.

The galectin-3 levels were higher in the COVpos group than they were in the COVneg group (Figure 1A). COVpos patients also exhibited higher levels of vWF (Figure 1B), antithrombin (Figure 1D) and tPA (Figure 1E). No significant differences between the groups were observed in relation to p-selectin (Figure 1C) or D-dimer (Figure 1F).

Galectin-3 correlated significantly with vWF and mean platelet volume (MPV) as indicators of platelet activation and D-dimer as a marker of plasmatic coagulation in the COVpos group, but not in the COVneg group. No positive correlation existed between galectin-3 and p-selectin or tPA. When we were analyzing the association between galectin-3 and antithrombin, a positive correlation in the whole population was observed (Table 2).

### 2.3. Levels of Markers Associated with Treg Are Increased in COVID-19 and Correlate with Galectin-3 Levels

**Figure 2:**
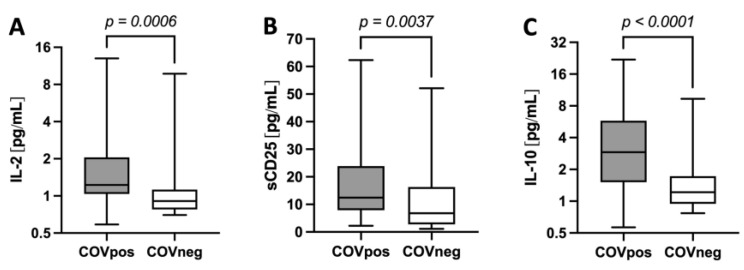
Levels of markers associated with Treg such as IL-2 (**A**), sCD25 or IL-2R (**B**) and IL-10 (**C**) in COVpos (n = 55) and COVneg (n = 35) patients.

The levels of interleukin IL-2 (Figure 2A), sCD25 (IL-2 receptor, Figure 2B) and IL-10 (Figure 2C) were higher in the COVpos than they were the COVneg patients.

Galectin 3 correlated with these markers in the COVpos group, but not with those in the COVneg cohort (Table 3).

### 2.4. Galectin-3 Levels Relate to Clinical Severity

**Figure 3:**
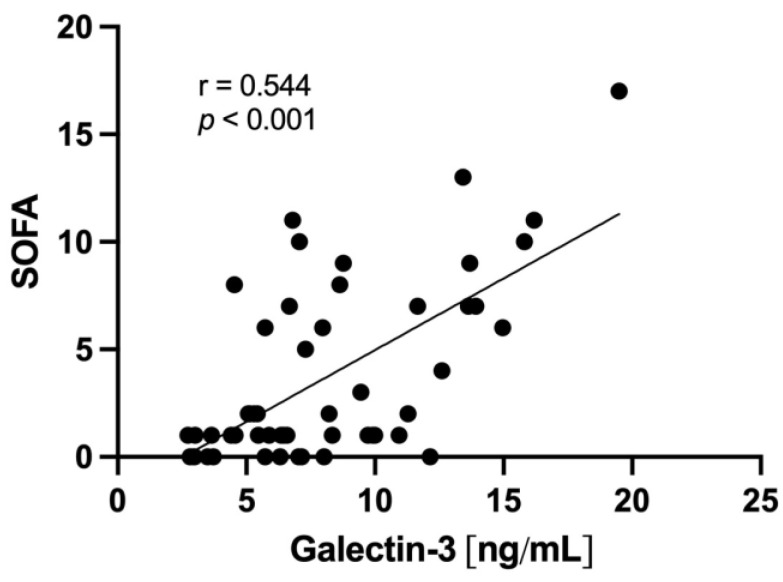
Correlation of galectin-3 levels and SOFA Score in the general population (n = 52).

The SOFA Score as a marker of clinical disease severity at the time of inclusion was higher in the COVpos than in the COVneg cohort (median 6 [1–10] vs. 1 [0–1.75]). A positive correlation between galectin-3 and the SOFA Score was observed in the total population (r = 0.544; *p* < 0.001) and in both COVpos (r = 0.430; *p* = 0.022) and COVneg (r = 0.609; *p* = 0.002) groups (Figure 3).

## 3. Discussion

In the present study, we assessed galectin-3 levels, as well as distinct coagulation and immune reaction markers, in a cohort of COVID-19 patients and compared them with a cohort of patients with acute respiratory diseases other than COVID-19.

First, we observed that galectin-3 levels were higher in the COVpos than they were in the COVneg group. Galectin-3 is a protein with pleiotropic effects on different cellular functions, including modulation of the innate immune system, fibrosis and on the development of cardiovascular disease including atherosclerosis [17,39]. Our findings are consistent with previous studies, which have also described galectin-3 as a possible prognostic and severity marker in COVID-19 [22,40,41,42]. The difference between our cohort and populations from previous studies is mainly the control group. Most previous studies compared COVID-19 patients to mostly healthy populations. We have therefore used a control group of diseased patients with an acute respiratory infection, but without COVID-19, who were recruited during the same time as our COVID-19 patients to account for structural confounders in health care during this period. Interestingly, despite the fact that heart failure was more common in our control group and that galectin-3 is known as a marker for heart failure [43], its levels were even higher in the COVID-19 group that had a lower prevalence of heart failure.

Another finding of our study was that the levels of vWF as a marker for increased platelet activation, as well as other coagulation markers, were higher in the COVpos than they were in the COVneg group. vWF has previously been reported to be increased in COVID-19 patients and is associated with disease severity [44,45,46]. Regarding P-selectin, most previous studies showed that the levels of it are also increased in COVID-19 patients. P-selectin plays a role in the endothelial activation and leucocyte recruitment, which contributes to disease severity [9]. Notably, previous studies evaluating P-selectin levels have mostly included healthy subjects as the control group. However, other study results are conflicting and have also found no differences in the P-selectin levels between COVID-19 patients and controls [47]. Our data are in line with those of another study that also used diseased patients as controls [48].

The elevation of other coagulation markers in the COVpos group is consistent with reports from previous studies [5,16,49,50]. A trend towards increased D-dimer levels in COVID-19 patients was observed in our study, without reaching a statistically significant difference. This might also be due to the control group consisting of subjects with infectious diseases associated with activated and consumptive coagulation systems [51,52]. This underlines the importance of using a control group with patients with acute respiratory infections other than SARS-CoV-2.

In our study, we observed that galectin-3 and the above-mentioned platelet reactivity and coagulation markers correlated in COVpos patients, but not COVneg patients. Although this correlation was slightly weak, it was statistically significant. It has recently been reported that galectin-3 might serve as a thrombosis enhancer [28]. The fact that this correlation with platelet and coagulation markers was not present in the COVneg group points to a role of galectin-3 in the thrombogenic processes in COVID-19, and moreover, may have less relevance in non-COVID-19 respiratory infections. This is consistent with observations from the DEFINE Trial, in which COVID-19 patients were treated with an inhaled galectin-3 inhibitor. Here the authors observed that the inhibitor lowered the D-dimer level in comparison to that of the controls [53], which further suggests that galectin-3 inhibition can influence coagulopathy in COVID-19 patients.

We observed that the levels of markers that are associated with Tregs (IL-2, IL-10 and sCD25) were higher in the COVpos than they were in the COVneg group. This is consistent with previous data that have shown that, in particular, IL-10 and IL-2 levels are higher in COVID-19 patients and associated with disease severity [32,33,37,38]. We also observed that galectin-3 correlated weakly, but statistically significantly, with the above-mentioned interleukines in the COVpos group, but not in the COVneg group [36]. In 2021, Shahneh et al. described how Treg cells accumulated in thrombi, contributing to blood clot resolution [30]. Moreover, Treg cell dysregulation is linked to the alterations in long COVID [54]. Thus, our and the findings of others raise the question of whether a possible relation between galectin-3 and the development of post-acute SARS-CoV-2 sequelae might exist.

The link between galectin-3 and clinical severity, as observed in our cohort, in both COVpos and COVneg patients supports findings from previous studies on COVID-19 [22,40]. The notion that this positive correlation was also observed in the COVneg group may be due to the fact that our controls were not healthy subjects, but patients with an acute respiratory infection as well. The evaluation of underlying molecular pathomechanisms by which galectin-3 influences hypercoagulability is a topic of future research.

### Limitations

Our study included patients from the beginning of the pandemic, and for this reason, the patients were not vaccinated. Only one of the patients received immunomodulatory therapy, and no information was available about the virus strain. Nowadays, the cellular immune response and cytokine profile of patients with COVID-19 is different due to vaccines and available antiviral therapies. Lastly, since it was a cross-sectional study without a follow-up, it was not possible to correlate the measured markers with the clinical outcomes of thrombotic events.

## 4. Materials and Methods

### 4.1. Study Design and Study Population

In this observational study, we included 90 patients who were hospitalized (either on a normal ward or ICU) due to acute respiratory infections between May 2020 and May 2021 in Charité—Universitätsmedizin Berlin, Germany. The study was approved by the local Ethics Committee (EA2/066/20, EA4/147/15) and was conducted in accordance with the ethical standards of the Helsinki Declaration of 1975. Fifty-five patients were confirmed to be SARS-CoV-2 positive by a positive polymerase chain reaction (PCR) test. The control group consisted of 35 patients who were SARS-CoV-2 negative. All study subjects had to be at least 18 years old. Patients with hematological diseases, coagulopathies or who experienced acute bleeding events, as well as those having undergone dual antiplatelet therapy, were excluded from study participation.

### 4.2. Data Collection

Routine laboratory data (creatinine, blood urea nitrogen, NT-proBNP, C-reactive protein, hemoglobin, leucocyte and thrombocyte count and MPV) were measured in the local laboratory. The baseline characteristics were taken from the hospital electronic medical records. Blood samples from all patients were collected by venous puncturing, and plasma was separated and stored at −80 °C. The SOFA Score at the time of inclusion was calculated in the patients for whom the required parameters were available (52 subjects).

### 4.3. Enzyme-Linked Immunosorbend Assay

To study galectin-3 (R&D Systems, DGAL30, Minneapolis, MN, USA), von Willebrand Factor (vWF) (AssayPro, EV2030-1, Saint Charles, MO, USA), p-selectin (Cloud-Clone, SEA569Hu, TX, USA), tPA (AssayPro, ET2001-7, Saint Charles, MO, USA) and D-dimer (Diagnostica, Asserachrom D-Di 00947), an enzyme-linked immunosorbent assay (ELISA) was performed according to the manufacturer’s instructions and measured with the Tecan Infinite 200Pro plate reader (Tecan Group, Maennedorf, Switzerland).

### 4.4. Multiplex Bead-Based Arrays

Cytokines IL-2 and IL-10 were measured using the “COVID-19 Cytokine Storm Panel-1”, and cytokines IL-13 and sCD25 were measured using the “COVID-19 Cytokine Storm Panel-2”. These are multiplex, bead-based arrays (BioLegend 741091 and 741142, San Diego, CA, USA). Antithrombin levels were measured using the “Human Fibrinolysis Panel”, which is also a multiplex, bead-based array (BioLegend, 740761, CA, USA). The data were measured with the Attune NxT Acoustic Focusing Cytometer (ThermoFisherScientific, Waltham, MA, USA) and analyzed using the Kaluza version 2.1 software.

### 4.5. Statistical Analysis

Continuous variables are presented as medians with interquartile ranges, and the categorical variables are presented as frequencies and percentages. To evaluate the differences between two groups, Mann–Whitney U test or Fisher´s exact test was used, respectively. To analyze the correlation between continuous variables, Spearman´s correlation was calculated. All tests were two-sided ones; 95% confidence intervals were used, and a *p*-value < 0.05 was considered to be statistically significant. Statistical analyses were performed using the software IBM SPSS Statistics version 28.0.1, and the graphs were generated with GraphPad Prism 9.5.

## 5. Conclusions

In COVID-19, but not in other acute respiratory diseases, galectin-3 is associated with platelet activation markers and increased thrombogenicity markers. Galectin-3 is also associated with disease severity, which suggests that it could be a potential treatment target in patients with COVID-19.

## Figures and Tables

**Table 1:** Baseline characteristics.

	Total Population (n = 90)	COVpos (n = 55)	COVneg (n = 35)	*p* Value
Age	70 (56.5–79.25)	69 (55–76)	73 (58–81)	0.146
Gender, male	58 (64.4%)	38 (69.1%)	20 (57.1%)	0.267
BMI (kg/m^2^) *	26.33 (24.58–30.33)	26.70 (24.69–30.70)	25.11 (22.92–28-50)	0.135
On ICU	26 (28.9%)	20 (36.4%)	6 (17.1%)	0.059
Died	10 (11.1%)	10 (18.2%)	0 (0%)	0.006
**Pre-existing conditions**				
Heart failure	9 (10%)	2 (3.6%)	7 (20%)	0.025
Coronary artery disease	19 (21.1%)	7 (12.7%)	12 (34.3%)	0.019
Arterial hypertension	58 (64.4%)	34 (61.8%)	24 (68.6%)	0.652
Diabetes mellitus	22 (24.4%)	14 (25.5%)	8 (22.9%)	1.0
Peripheral artery disease	14 (15.6%)	2 (3.6%)	12 (34.3%)	<0.001
Hypercholesterinemia	25 (27.8%)	15 (27.3%)	10 (28.6%)	1.0
COPD	17 (18.9%)	4 (7.3%)	13 (37.1%)	<0.001
**Medication**				
Prophylactic anticoagulation	56 (62.2%)	34 (61.8%)	22 (62.9%)	1.0
Therapeutic anticoagulation	34 (37.8%)	21 (38.2%)	13 (37.1%)	1.0
ASS	35 (38.9%)	23 (41.8%)	12 (34.3%)	0.513
ADP Receptor Antagonist	2 (2.2%)	2 (3.6%)	0 (0%)	0.519
Betablocker	32 (35.6%)	15 (27.3%)	17 (48.6%)	0.046
RAAS-blockage	35 (38.9%)	17 (30.9%)	18 (51.4%)	0.076
Diuretics	36 (40%)	19 (34.5%)	17 (48.6)	0.195
Statins	23 (25.6%)	14 (25.5%)	9 (25.7%)	1.0
Glucocorticoids	39 (43.3%)	32 (58.2%)	7 (20%)	<0.001
Remdesivir	1 (1.1%)	1 (1.8%)	0 (0%)	1.0
Tocilizumab	1 (1.1%)	1 (1.8%)	0 (0%)	1.0
Inhalative Therapy	63 (70%)	41 (74.5%)	22 (62.9%)	0.250
**Laboratory values**				
Creatinine (mg/dL) *	0.92 (0.76–1.19)	0.92 (0.7–1.16)	0.94 (0.78–1.23)	0.649
BUN (mg/dL) *	41.5 (27–59)	48 (27–63)	34 (25–52)	0.105
NT-proBNP (ng/L) *	488 (173–1695.25)	359 (136–1616)	930 (322–2318)	0.079
CRP (mg/dL) *	65.3 (25.6–105.05)	69 (18.9–125)	62.1 (38.78–102.15)	0.807
Hemoglobin (g/dL) *	11.5 (9.78–12.8)	11.4 (9.4–12.6)	11.8 (10.1 -13.4)	0.204
Leukocytes (n/nL) *	8.77 (6.72–12.16)	8.51 (6.77–12.37)	8.83 (6.67–11.16)	0.878
Thrombocytes (n/pL) *	281.5 (229.25–371.25)	297 (234–397)	279 (209–322)	0.111
MPV (fL) *	10.4 (9.7–11.23)	10.4 (9.68–11.53)	10.35 (9.7–10.98)	0.374

* Continuous values are presented as median and interquartile range. BMI: body mass index, ICU: intensive care unit; COPD: chronic obstructive pulmonary disease; ASS: aspirin; ADP: adenosine diphosphate; RAAS: renin-angiotensin system; BUN: blood urea nitrogen; NT-proBNP: N-terminal prohormone of brain natriuretic peptide; CRP: C-reactive protein; MPV: mean platelet volume.

**Table 2:** Correlations between galectin-3 and markers of increased thrombogenicity.

	Galectin-3 (ng/mL)					
	Total Population		COVpos		COVneg	
vWF (IU/mL)	r = 0.380	***p* < 0.001**	r = 0.428	***p* = 0.002**	r = 0.122	*p* = 0.485
MPV (fL)	r = 0.212	*p* = 0.054	r = 0.346	***p* = 0.011**	r = −0.057	*p* = 0.757
P-selectin (ng/mL)	r = −0.129	*p* = 0.241	r = 0.000	*p* = 1.000	r = −0.241	*p* = 0.162
Antithrombin (ng/mL)	r = 0.432	***p* < 0.001**	r = 0.339	***p* = 0.014**	r = 0.430	***p* = 0.010**
tPA (ng/mL)	r = 0.015	*p* = 0.896	r = 0.028	*p* = 0.846	r = −0.294	*p* = 0.114
D-dimer (mg/mL)	r = 0.385	***p* < 0.001**	r = 0.462	***p* < 0.001**	r = 0.196	*p* = 0.267

vWF: von Willebrand factor; MPV: mean platelet volume; tPA: tissue plasminogen activator.

**Table 3:** Correlations between galectin-3 and markers of T cell activation.

	Galectin-3 (ng/mL)					
	Total Population		COVpos		COVneg	
IL-2 (pg/mL)	r = 0.181	*p* = 0.105	r = 0.308	***p* = 0.037**	r = −0.032	*p* = 0.854
IL-10 (pg/mL)	r = 0.258	***p* = 0.016**	r = 0.336	***p* = 0.015**	r = −0.093	*p* = 0.594
sCD25 (pg/mL)	r = 0.346	***p* = 0.001**	r = 0.392	***p* = 0.004**	r = 0.183	*p* = 0.293

## Data Availability

Data from patients are not publicly available due to general data protection regulations. They could be made available upon request.
